# Unconstrained one-stage total knee arthroplasty PS design in patient with secondary osteoarthritis due to granulomatous infection with medial femoral condyle defect: A case report

**DOI:** 10.1016/j.ijscr.2022.107469

**Published:** 2022-07-30

**Authors:** Nur Rahmansyah, Dicky Mulyadi, Raden Moechammad Satrio Nugroho Magetsari, Aditya Fuad Robby Triangga

**Affiliations:** a Lecturer of Medical Faculty of Bosowa University, Makassar, Indonesia; b Department of Orthopedics and Traumatology, Dr. La Palaloi General Hospital, Maros, Indonesia; c Department of Orthopaedic and Traumatology, Faculty of Medicine, Padjajaran University, Bandung, Indonesia; d Division of Adult Reconstructive Surgery and Sports Injury, Dr.Hasan Sadikin General Hospital, Bandung, Indonesia; e Department of Orthopaedic and Traumatology, Faculty of Medicine, Public Health and Nursing, Universitas Gadjah Mada/Dr. Sardjito Hospital, Yogyakarta, Indonesia; f Division of Adult Reconstructive Surgery and Sports Injury, Dr. Sardjito Hospital General Hospital, Yogyakarta, Indonesia

**Keywords:** Granulomatous infection, *mycobacterium tuberculosis*, Secondary osteoarthritis, Unconstrained tka, One stage tka, Case report

## Abstract

**Introduction and importance:**

Granulomatous *Mycobacterium Tuberculosis* Infection Causes Secondary Knee Osteoarthritis is still a point of contention in terms of therapy, whether it is done early in the first stage or later in the second stage of knee surgery. Early Total Knee Arthroplasty as a therapy for secondary knee osteoarthritis induced by Granulomatous *mycobacterium tuberculosis* infection is still performed rarely.

**Case presentation:**

A case of left pain and swollen knee in males for 8 months. Because of pain and reduced knee range of motion, the patient now has an antalgic gait, which make him difficult to do daily activities. Treatment with medications and physiotherapy failed. Radiographs revealed juxta-articular osteoporosis, peripherally distributed osseous erosions, joint space narrowing, and a bony defect in the medial femoral condyle. This case was successfully treated using Unconstrained Knee Arthroplasty PS Design.

**Clinical discussion:**

Case selection for granulomatous infection case is key element to determine whether a single TKA procedure can be used to treat knee pain problems as a result of secondary osteoarthritis.

**Conclusion:**

This case shows secondary knee osteoarthritis caused by Granulomatous *Mycobacterium Tuberculosis* Infection without pyogenic pus production might allow for early one-stage total knee arthroplasty. Three months following surgery, the patient's knee was stable and painless, with good wound healing and no signs of infection.

## Highlights

• Granulomatous *Mycobacterium Tuberculosis* Infection Knee Osteoarthritis is still a point of contention in decision of one or two stage knee arthroplasty surgery
• Case selection is the most important to determine whether the patient is proper for one stage total knee arthroplasty in patient granulomatous knee infection
• This case showed good outcome 3 month following single stage total knee arthroplasty in in patient granulomatous knee infection without pus formation.

## 1 Introduction and importance

Infections of both the bone or joints that manifest histopathological as granulomas can be classified as having bacterial or fungal aetiology [1]. A granuloma is an inflammatory mononuclear cell infiltration that, although capable of inhibiting *Mycobacterium tuberculosis* development, also provides a survival environment from which the bacteria can spread [2]. The *Mycobacterium tuberculosis* lesion is very dynamic, driven by both immune response components and the pathogen [2].

Tuberculosis (TB) has historically been known as a disease with numerous clinical presentations that frequently lead to misdiagnosis [3]. According to the World Health Organization (WHO), Indonesia has the third-highest prevalence of TB in 2019. Indonesia was also ranked one of the ten countries with the greatest differential between the number of new cases reported and the 10 million incident cases recorded in 2018 [4]. Extra-pulmonary TB (Tuberculosis) accounted for more than a third of all TB cases in China, with skeletal and pleural symptoms having the highest incidence [5]. Skeletal TB manifestations represented about a third of all extra-pulmonary symptoms, with the largest prevalence presenting in the spine, hip, and knee [6]. This disease's mimicking presentation makes diagnosis challenging. It is particularly common in extrapulmonary tuberculosis. [7]. As a result, this condition is frequently exhibited as acute infection and malignancy, resulting in delayed or underdiagnosis and medical therapy.

Tuberculosis of the knee has often resulted in significant bone loss by the time it is clearly detected due to a lack of clinical features indications [8], [9]. Advanced tuberculous arthritis is characterized as a clinical presentation of a restricted range of motion, as well as radiological signs of considerable reduction of joint space, alteration of joint surfaces, or even joint subluxation or dislocation [10]. Arthrodesis, resection arthroplasty, and total knee arthroplasty (TKA) are all surgical procedures for advanced tuberculous arthritis [10], [11], [12]. While they provide pain relief and infection management, the first two procedures frequently result in decreased limb function and potential consequences [11], [12]. TKA is believed to be a successful treatment, although there are controversies on operation time, surgical planning, and perioperative therapeutic regimens for advanced tuberculosis [13], [14], [15], [16], [17].

In this paper, we presented a 51-year-old man with unilateral left knee pain, edema, and warmth that had gone undiagnosed for 8 months. The work has been reported in line with the SCARE 2020 criteria [18].

## 2 Case presentation

A 51-year-old Sundanese Craftsman was sent to the orthopaedic clinic by his family physician for pain in his left knee. His complaints had progressively deteriorated over eight months, to the point that he now had an antalgic gait, makes it hard to climb stairs, and had a limited range of motion in his knee. Painkillers drug and physiotherapy had made no difference to reduce his pain. There is no recent history of trauma, respiratory, infectious, previous surgery, or joint problems. During this period of knee pain, the patient lost 10 kg body weight. He is a smoker and comes from low socioeconomic level.

**Fig. 1:**
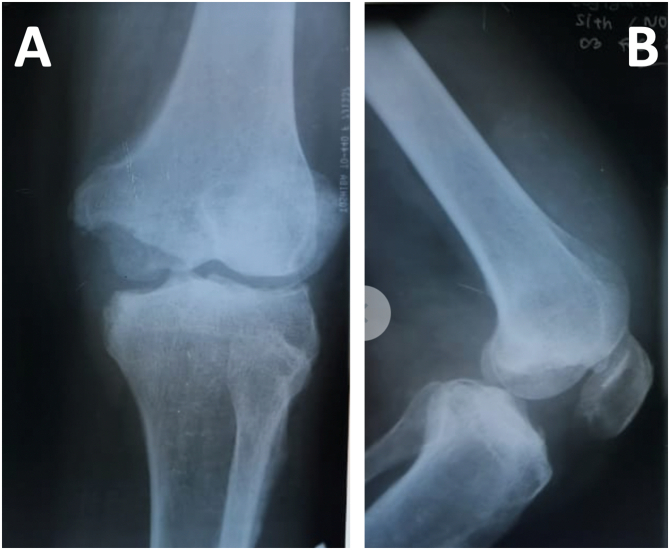
Preoperative Left Knee Radiograph Antero Posterior and Lateral View juxta-articular osteoporosis, peripherally distributed osseous erosions, joint space narrowing, and medial femoral condyle bony defect (A)(B).

**Fig. 2:**
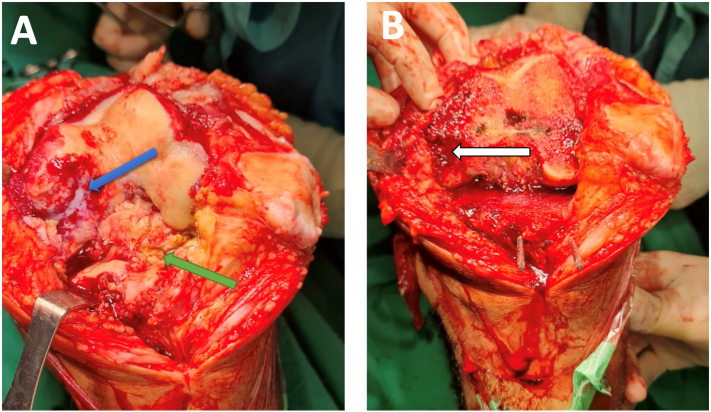
Intraoperative photograph showing the large bone defect of the left distal medial femoral condyle (blue arrow) caseous tissue necrosis (green arrow) (A), minimal bone defect after proper bone cut distal femur (white arrow) (B).

The left knee was shown to be swollen and swollen, with a fixed flexion contracture of 10°, flexion to 80°, and also the knee held in a varus of around 15°. The left hip, left ankle, and right knee were all found to be normal. A chest radiograph was normal, but radiographs of the left knee (Fig. 1, Fig. 2) juxta-articular osteoporosis, peripherally distributed osseous erosions, joint space narrowing, and medial femoral condyle bony defect (Fig. 1A–B). Blood testing showed elevated C-reactive protein (CRP) of 5.4 mg/dL (normal 0-5 mg/L), erythrocyte sedimentation rate (ESR) of 38 mm/h (normal 1–10 mm/h), and white blood cell count of 11.0 × 10^9^/L.

The patient was scheduled for arthrotomy and early total knee arthroplasty if there is no sign of pyogenic infection intraoperative after screening of optimal general condition and receiving informed consent regarding the surgical technique and potential risks.

**Fig. 3:**
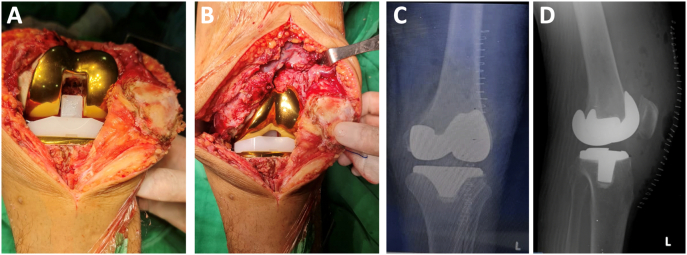
Intraoperative photograph after insertion Unconstrained PS Design Knee Implant (A)(B), Post-operative Xray Anteroposterior Lateral View (C)(D).

The surgery was conducted in the supine position under spinal anaesthesia. Surgery was performed, by DM an experienced hip & knee surgeon, arthrotomy with medial parapatellar approach. The patella dislocated laterally, synovial fluid look good without pus and sign infection, there were found tissue like caseous necrosis suspicious granulomatous infection *Mycobacterium tuberculosis*, and medial femoral condyle defect. Adequate arthrotomy to remove tissue necrosis and proper bone cutting to convert large bone defects to be minimal bone defects (Fig. 2A–B). Acid-fast bacilli specimens were needed, as well as specimens for microscopy, culture, and sensitivity. After adequate removal of tissue necrosis, Unconstrained PS Design Total Knee Arthroplasty performed with Femoral stem size 3 Left (ACS® PS Femoral Component Cemented, Implant Cast, Germany), Tibial stem size 3.5 Left (ACS® FB Tibial Component Cemented, Implant Cast, Germany), Tibial insert 12,5 mm (ACS® FB PS PE-Insert Hyperflex, Implant Cast, Germany) (Fig. 3A–B).

**Fig. 4:**
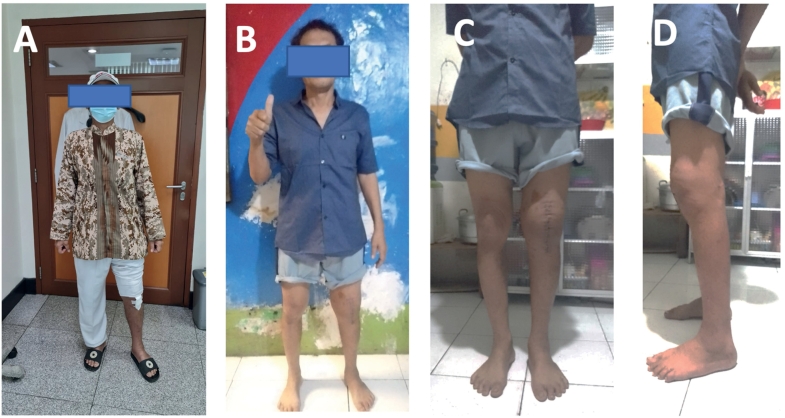
Two weeks following surgery (A). 3 months follow-up after One-Stage Unconstrained TKA (B)(C)(D).

Following surgery, The patient received postoperative management including cephalosporin antibiotics, ketorolac injections, and routine wound care. A standardized rehabilitation procedure was carried out on the patients. Synovial fluid cultures were found to have developed Mycobacterium TB after twelve days. All usual anti-tuberculosis drugs were effective against the bacteria. The histopathologic examination revealed granulomatous inflammation with caseous necrosis, suggesting tuberculosis. The patient was referred to a pulmonologist. The wound healing well, and the patient was able to walk without assistance 3 months following surgery to do daily activities. (Fig. 4A–D). The patient feel happy after the operation after eight month full of pain wherever he went and he could his daily activities as a craftsman to make his family could life.

## 3 Clinical discussion

*Mycobacterium tuberculosis* identification is required for a conclusive diagnosis of TB arthritis based on culture or histopathologic tests. Only 0 to 5 % of patients have a positive acid-fast stain test, whereas *M. tuberculosis* is identified in 60 to 90 % of cases [19].

Medical treatment for tuberculosis is always based on a combination therapy, which is divided into two phases: the first phase (typically based on four medications) lasts two months, and the continuation phase (generally based on two drugs) can last from four to seven months or longer in some situations [20], [21]. The best length of treatment for musculoskeletal tuberculosis is unknown. The majority of patients require treatment for 6 to 9 months [22], but individuals with severe or advanced illness may require longer (9–12 months) [23].

When the TB in adult patients' knees is limited to the bones, the most usual therapy is arthrotomy and curettage of the lesion. Complete debridement is useful for effective TB arthritis therapy, as it can remove diseased tissues and reduce the chance of disease recurrence. The greatest treatment option in the past was articular debridement and joint fusion, but postoperative joint function was poor, which had a major impact on patients' working and daily life activities. A recurrence rate of 1 to 9 % has also been recorded. Unfortunately, joint fusion is frequently the only effective therapy option. Arthrodesis may be more appropriate for individuals who have less bone loss and a large cancellous surface, allowing for effective bone apposition and compression [24].

Total knee arthroplasty (TKA) can provide patients with pain relief while also restoring knee function. Before implanting a TKA, an attempt should be made to eliminate tuberculosis. Habaxi et al. [16], proposed delaying anti-TB medication until ESR levels began to fall, maybe to less than 40 mm/h. Additionally, surgeons should clean out the joint capsule and synovial tissue necrosis, as well as check for sinuses. Some writers suggest that cemented TKA should be used to repair potential bone deficiencies in knee TB. Furthermore, the generation of heat during bone cement polymerization can kill Mycobacterium and reduce the likelihood of recurrence [16].

Several studies [25], [26], [27] on the adherence and production of small membrane-like material on the fixture or artificial prosthesis, including *mycobacterium tuberculosis*, provide a reliable theoretical basis for prosthesis implantation. The choice of prosthesis is equally critical. Some experts feel that TKA inactive TB of the knee joint should employ a cemented prosthesis because the heat produced by bone cement polymerization can kill *Mycobacterium tuberculosis* and reduce the incidence of recurrence. Cemented prosthesis is the best option for individuals with bone defects.

In this case patient with knee pain, Xray examination showed juxta-articular osteoporosis, peripherally distributed osseous erosions, joint space narrowing, and medial femoral condyle bony defect, laboratory examination Erytrosit Sedimentation Rate 38 mm/h, white blood cell count of 11.0 × 10^9^/L performed cemented unconstrained total knee arthroplasty as management of Tuberculosis infection knee with bone defect with good outcome.

## 4 Conclusion

As a treatment for secondary knee arthritis caused by granulomatous infection *Mycobacterium Tuberculosis*, one-stage total knee arthroplasty poses significant challenges and remains controversial. Case selection is critical to avoid misdiagnosis as pyogenic infection or *Mycobacterium Tuberculosis*.

In this case, management bone defect total knee arthroplasty for Tuberculosis was performed in this case. After 3 months, a knee with a bone defect has a stable result, and a non-constrained implant works well. More in-depth study and long-term follow-up reports are required.

## Source of funding

None.

## Ethical approval

Our institutional review board does not provide an ethical approval in the form of case report/case series.

## Consent

Written informed consent was given before any procedures are undertaken.

## Guarantor

Dicky Mulyadi.

## Provenance and peer review

Not commissioned, externally peer-reviewed.

## CRediT authorship contribution statement

Nur Rahmansyah (NS): Writing the paper, English checking, Asistent Surgeon.

Dicky Mulyadi (DM): Surgeon, conceptualization, and writing the paper.

Raden Moechammad Satrio Nugroho Magetsari (SNM): Editing, English checking and manuscript reviewing.

Aditya Fuad Robby Triangga (RBY): Editing, English checking, and manuscript reviewing.

All authors discussed the results and contributed to the final manuscript.

## Declaration of competing interest

The authors declare that there are no conflicts of interest regarding the publication of this article.

## References

[1] Pritchard D.J. (1975 Oct). Granulomatous infections of bones and joints. Orthop. Clin. North Am..

[2] Ehlers S., Schaible U.E. (2012). The granuloma in tuberculosis: dynamics of a host-pathogen collusion. Front. Immunol..

[3] Kulchavenya E. (2014 Apr 1). Extrapulmonary tuberculosis: are statistical reports accurate?. Ther. Adv. Infect. Dis..

[4] (2019).

[5] Pang Y., An J., Shu W., Huo F., Chu N., Gao M. (2019 Mar 1). Epidemiology of extrapulmonary tuberculosis among inpatients, China, 2008–2017 - volume 25, number 3—March 2019 - emerging infectious diseases journal - CDC. Emerg. Infect. Dis..

[6] Peto H.M., Pratt R.H., Harrington T.A., LoBue P.A., Armstrong L.R. (2009 Nov). Epidemiology of extrapulmonary tuberculosis in the United States, 1993–2006. Clin. Infect. Dis..

[7] Lidder S., Lang K., Haroon M., Shahidi M., El-Guindi M. (2009 Oct 10). Tuberculosis of the knee. Orthop. Rev. (Pavia).

[8] Wolfgang G.L. (1985 Dec). Tuberculosis joint infection following total knee arthroplasty. Clin. Orthop. Relat. Res..

[9] Watts H.G., Lifeso R.M. (1996 Feb). Tuberculosis of bones and joints. J. Bone Joint Surg. Am..

[10] Tuli S.M. (2002 May). General principles of osteoarticular tuberculosis. Clin. Orthop. Relat. Res..

[11] Bae D.K., Yoon K.H., Kim H.S., Song S.J. (2005 Mar). Total knee arthroplasty in stiff knees after previous infection. J. Bone Joint Surg. Br..

[12] Lim H.C., Bae J.H., Hur C.R., Oh J.K., Han S.H. (2009 Feb 1). Arthrodesis of the knee using cannulated screws. J. Bone Joint Surg. B.

[13] Eskola A., Santavirta S., Konttinen Y.T., Tallroth K., Lindholm S.T. (1988 Nov). Arthroplasty for old tuberculosis of the knee. J. Bone Joint Surg. Br..

[14] Kim Y.H. (1988 Oct). Total knee arthroplasty for tuberculous arthritis. J. Bone Joint Surg. Am..

[15] Su J.Y., Huang T.L., Lin S.Y. (1996 Feb). Total knee arthroplasty in tuberculous arthritis. Clin. Orthop. Relat. Res..

[16] Habaxi K.K., Wang L., Miao X.G., Alimasi W.Q.K., Zhao X.B., Su J.G. (2014). Total knee arthroplasty treatment of active tuberculosis of the knee: a review of 10 cases. Eur. Rev. Med. Pharmacol. Sci..

[17] Oztürkmen Y., Uzümcügil O., Karamehmetoğlu M., Leblebici C., Caniklioğlu M. (2014 May). Total knee arthroplasty for the management of joint destruction in tuberculous arthritis. Knee Surg. Sports Traumatol. Arthrosc..

[18] Agha R.A., Franchi T., Sohrabi C., Mathew G., Kerwan A., SCARE Group (2020 Dec). The SCARE 2020 guideline: updating consensus Surgical CAse REport (SCARE) guidelines. Int J Surg..

[19] Hopewell P.C. (2014 Apr). Overview of clinical tuberculosis. Tuberculosis.

[20] Blumberg H.M., Burman W.J., Chaisson R.E., Daley C.L., Etkind S.C., Friedman L.N. (2003). American Thoracic Society/Centers for Disease Control and Prevention/Infectious Diseases Society of America: treatment of tuberculosis. Am. J. Respir. Crit. Care Med..

[21] Hopewell P.C., Pai M., Maher D., Uplekar M., Raviglione M.C. (2006 Nov). International standards for tuberculosis care. Lancet Infect. Dis..

[22] CDC, American Thoracic Society, Infectious Diseases Society of America (2003). Treatment of tuberculosis. MMWR Recomm Rep..

[23] Blumberg H.M., Leonard M.K., Jasmer R.M. (2005 Jun 8). Update on the treatment of tuberculosis and latent tuberculosis infection. JAMA.

[24] Tang X., Zhu J., Li Q., Chen G., Fu W., Li J. (2015 Aug 19). Knee arthrodesis using a unilateral external fixator combined with crossed cannulated screws for the treatment of end-stage tuberculosis of the knee. BMC Musculoskelet. Disord..

[25] Saunders B.M., Frank A.A., Orme I.M. (1999). Granuloma formation is required to contain bacillus growth and delay mortality in mice chronically infected with mycobacterium tuberculosis. Immunology.

[26] Marmor M., Parnes N., Dekel S. (2004). Tuberculosis infection complicating total knee arthroplasty: report of 3 cases and review of the literature. J. Arthroplast..

[27] Ha K.Y., Chung Y.G., Ryoo S.J. (2005). Adherence and biofilm formation of Staphylococcus epidermidis and Mycobacterium tuberculosis on various spinal implants. Spine (Phila Pa 1976).

